# Pre-approval incentives to promote adoption of personalized medicine: a theoretical approach

**DOI:** 10.1186/s13561-019-0244-8

**Published:** 2019-10-29

**Authors:** F. Antoñanzas, C. A. Juárez-Castelló, R. Rodríguez-Ibeas

**Affiliations:** Department of Economics, University of La Rioja, 26004 Logroño, Spain

**Keywords:** Personalized medicine, Biomarker, Pay-for-performance, Price and reimbursement policy

## Abstract

**Background:**

Currently, personalised medicine is becoming more frequently used and many drug companies are including this strategy to gain market access for very specialized therapies. In this article, in order to understand the relationships between the health authority and the drug company when deciding upon the implementation of personalized medicines, we take a theoretical perspective to model it when the price and reimbursement policy follows a pay-for-performance scheme. During the development of a new drug, the firm must decide whether to generate additional knowledge by investing in additional resources to stratify the target population based on a biomarker or directly apply for marketing authorization for the new treatment without information on the characteristics of patients who could respond to it. In this context, we assume that the pricing policy is set by the health authority, and then we characterize the pricing and investment decisions contingent on the rate of response to the treatment.

**Results:**

We find that the price when the firm carries out R&D leading to the personalized treatments is not necessarily higher than the price if the firm does not carry out the R&D investment. When the rate of response to the treatment is too low, then the new drug is not marketed. If the rate of response is too high, personalized medicine is not implemented. For intermediate values of the rate of response, the adoption of personalized medicine may occur if the investment costs are sufficiently low; otherwise, the treatment is given to all patients without additional information on their characteristics. The higher the quality of the genetic test (in terms of its sensitivity and specificity), the wider the interval for the values of the proportional responders for which personalized medicine may be implemented.

**Conclusions:**

Our findings show that pre-approval incentives (prices) to promote the personalized treatments depend on the specific characteristics of the disease and the efficacy of the treatment. The model gives an intuitive idea about what to expect in terms of price incentives when the possibility of personalizing treatments becomes a strategic decision for the stakeholders.

## Background

Health care systems continuously face the challenge of making the prescription of expensive but low-effectiveness drugs compatible with the financial sustainability of the system [1–3]. The new paradigm of personalized medicine strives to overcome this issue by tailoring therapies to patients who are thus expected to respond better. Biomarkers (genetic, biochemical or molecular) that can help predict clinical outcomes of a given treatment and hence inform the choice of therapy are used to stratify patients. For that purpose, additional R&D is required, mainly carried out by drug firms. These firms therefore have to make strategic decisions about making this additional effort before applying for approval of a new therapy or once a drug is already on the market [4, 5].

Implementing personalized medicine requires a biomarker to identify potential responders and a diagnostic test. The analysis of the situation in which the firm carries out the identification of the biomarker when the drug is already on the market (post-approval case) has already been studied as a way to mitigate the costs of managed entry agreements [6]. However, the conceptualization of the pre-approval decision to personalize treatment has not been addressed yet. In this case, the firm may invest in additional R&D to try to obtain a biomarker that will allow it to stratify the target population. Once the biomarker is found, the firm may develop a diagnostic test to identify patients who have the biomarker (companion diagnostic) and market the test and the drug together, or alternatively there may already be tests available on the market [7]. In the first case, the drug can only be administered to patients who have been tested with the companion diagnostic test, while in the latter, patients may be identified using other tests.

Until now, personalized medicine has been mainly applied in oncology. Many new drugs are added to or combined with existing ones following a treatment algorithm. For example, trastuzumab, approved in 1998, in combination with chemotherapy for human epidermal growth factor receptor 2 positive (HER2+) for early breast cancer; pertuzumab, approved in 2012, in combination with trastuzumab is indicated for metastatic breast cancer in HER2+; or panitumumab, approved in 2006, for first-line metastatic RAS/wild-type colorectal cancer in combination with FOLFIRI, for approximately 40% of patients. These are prescribed to the patients indicated to receive them [8–11], and they have increased survival rates over the last 15 years. The prescription of these drugs requires the patient to be previously screened by a genetic test. The pharmaceutical firms had to carry out the necessary research to identify the genetic mutation and the proper biomarker so that the new product be prescribed to the selected group of patients. A review, performed by the authors, of the last three years authorized cancer drugs by the European Medicines Agency shows that approximately half of them required patients to be screened by a genetic test before determining their treatment [12].

Firms anticipate, based on their experience, that a drug with a broad target population will be cheaper than one with a restricted subpopulation of patients. In the first case, a recent common approach to make the system sustainable and to facilitate easier access to a drug is to use a pay-for-performance agreement (see [13–17] for analytical models and guidelines on this subject). Alternatively, firms can implement an R&D process to find a biomarker that identifies best responders and hence restricts the target population, allowing them to market the drug at a higher price but with a smaller sales volume. This is the trade-off some pharmaceutical firms face nowadays, mainly in the oncology area where genomics has contributed to targeting new therapies.

On the other hand, health authorities face new challenges when making decisions on price and reimbursement for these treatments as they have to take into account factors such as the new incremental health outcomes for the selected group of patients –if identified–, the potential side effects if new drugs are administered to the whole patient population without stratification, and the usually increasing costs due to higher prices and longer treatment durations as some conditions become almost chronic for patients who progress slowly. At this point, the results of economic evaluations in this area provide some information about the efficiency of these new therapies [18–20].

In this article, we focus, from a theoretical perspective, on the relationship between a pharmaceutical company and a health authority when personalized treatments may be implemented. More specifically, we consider the situation in which a pharmaceutical firm with a new treatment, given the information on efficacy learned from Phase II and III clinical trials, must decide whether to carry out an R&D investment to generate additional knowledge to stratify the target population (e.g. to find a biomarker), potentially delaying the launching of the product, or to apply for marketing authorization. The firm may overlap the R&D programme with Phase II and III clinical trials to avoid postponing the launch of the treatment, or undertake the R&D investment after Phase III, delaying the launching of the treatment.

We treat the authorization rule as exogenous, and we focus only on the pricing and reimbursement policy set by a health authority. In particular, we assume that the treatment is authorized whenever it is expected to deliver net health benefits. We assume that the health authority follows a pay-for-performance mechanism if there is no stratification. We model the case in which the firm does not carry out the test directly but does identify the biomarker. Thus, we do not model the companion diagnostic decision. Both strategies have trade-offs in terms of price, benefits for the manufacturer and patients, as well as costs for the healthcare system. We characterize the decisions made by the firm and the health authority contingent on the efficacy of the treatment. This paper contributes to further understanding of the stakeholders’ decision-making processes concerning the adoption of personalized medicine.

The paper is organized as follows. In the next section we describe the model. In section 3, we characterize the behaviour of the pharmaceutical firm and the health authority, and determine the optimal pricing and reimbursement policy set by the health authority. In section 4 we discuss the main results of the analysis. Finally, we provide some conclusions and insights.

## Methods

We consider a population of patients suffering a disease whose size is normalized to 1. A firm has developed a new treatment for the disease. Let *δ* ∈ [0, 1] be the proportion of patients indicated to receive the new treatment (responders). We may alternatively interpret *δ* as the expected proportion or number of patients who benefit from the new treatment. The firm learns the value of *δ* after conducting Phase II and III clinical trials. For these patients, the efficacy of the treatment is 1, and the benefit is *B*. Patients who do not respond to the treatment have adverse effects *L*.^1^ This can be thought of as the existence of genetic characteristics differing between patients: some patients have a genetic factor while other patients lack it. Whether or not the patients respond to the new treatment depends on them having the genetic factor. The genetic factor can be identified by a predictive biomarker.^2^ A priori, the predictive biomarker is unknown by the firm.

We do not model the authorization policy, and we take it as exogenously given. In particular, we assume that the treatment is authorized whenever the expected health benefits from the Phase III clinical trial are non-negative: *δB* − (1 − *δ*)*L* ≥ 0. Hence, the treatment is authorized when the proportion of responders is above the threshold $\hat{\delta}=\frac{L}{B+L}$ . Thus, once the firm learns *δ*, it knows whether the treatment will be authorized or not.

When $\delta \ge \hat{\delta}$, the firm must decide between applying for authorization or undertaking an R&D investment to find the biomarker that will allow identification of responders by means of a diagnostic test. The cost of the R&D investment *I* is a random variable with probability distribution *F*(*I*) in the domain $\left[0,\overline{I}\right]$. In other words, R&D allows to identify the patients in the general population for whom the new treatment is indicated. We assume that once the biomarker is identified, there is a test available on the market (not co-developed with the companion new drug) that may be used to identify the patients with the biomarker.

Without loss of generality, we assume that there are a priori two potential biomarkers correlated with the genetic characteristics of the responders but the firm does not know which one can be used to identify the responders. For each biomarker, there is a test with specificity *e*_*i*_, sensitivity *s*_*i*_ and price *t*_*i*_, *i* = 1, 2. When the test *i* is administered, *s*_*i*_*δ* responders are identified and (1 − *e*_*i*_)(1 − *δ*) patients who are non-responders are identified as responders (false positives), *i* = 1, 2. Note that the test *i* correctly identifies *e*_*i*_(1 − *δ*) non-responders and (1 − *s*_*i*_)*δ* patients who are responders are identified as non-responders (false negatives), *i* = 1, 2. A priori, the probability that the biomarker *i* is associated to the genetic characteristics of the responders is *q*_*i*_ ∈ (0, 1), *i* = 1, 2. Let *s*, *e* and *t* denote respectively the expected values of the sensitivity, the specificity and the price. All this information is common knowledge.

The firm undertakes an R&D investment to know which biomarker is associated to the responders. The firm has private information about the cost of the R&D investment. After undertaking the R&D investment, the treatment is authorized, and the firm sells the treatment only to the patients identified as responders by the test. Patient stratification allows personalization of the treatment. If the firm does not undertake the R&D investment, the treatment is given to all patients as long as the expected health benefits net of payments to the firm are non-negative. Otherwise, the patients receive no treatment. We assume that non-treated patients experience neither benefits nor negative effects^3^ Note that if $\delta <\hat{\delta}$, the treatment is not authorized unless the firm undertakes R&D. The unit production cost of the new treatment is *c* ≥ 0. We assume *L* > *c*. The firm strives to maximize its profits.

The last agent in the model is the health authority which decides the pricing policy to maximize the expected health benefits net of payments to the firm. We assume that the firm is paid only if the patient is cured. In other words, the health authority follows a pay-for-performance policy with full penalization when the new treatment does not work. Without loss of generality, we will assume that there are no monitoring costs to determine whether the patient was cured or not. The pricing policy is contingent on the decision taken by the firm. Let *p*_*l*_ be the price when the firm does not undertake the R&D investment, and applies for authorization. Let *p*_*h*_ be the price when the patients, following the R&D investment, have been stratified. From the health authority’s perspective, the R&D investment reduces adverse effects. We assume that the health authority knows the distribution of the R&D investment cost but ignores the value of this variable when setting its pricing policy.

We have in mind the following situation. Given the pricing policy and the proportion of patients who respond to the new treatment learnt from the clinical trials, the firm must decide whether to search for additional information to select the target patient population (to identify the responders) or to apply for market authorization. If the firm follows the first strategy, the treatment is personalized, otherwise, the treatment is given to all patients. To simplify the analysis, we will assume that there are no launching delays if the firm researches new information to stratify the population of patients. We assume *L* > *t*, *e* + *s* > 1 and (*c* + *L*)(*B* − *c*)(*e* + *s* − 1) > *t*(*B* + *L*).^4^ The expected cost of the test must be lower than the adverse effects. Otherwise, paying for the test to avoid the adverse effects does not make sense. The last assumption is plausible as the price of the diagnostic tests in the real world is relatively low when compared to health benefits and adverse effects. We also assume that *t* < *s*(*B* − *c*) and *t* < *e*(*c* + *L*). In other words, *e* and *s* must be sufficiently high.

**Fig. 1:**
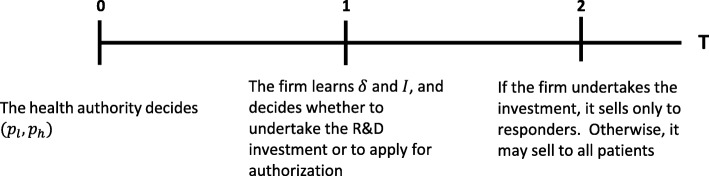
Timing of the model. T = time, *δ* = proportion of responders, I = the cost of the R&D investment, *p*_*l*_ = the price if the firm does not undertake R&D, *p*_*h*_ = the price if the firm undertakes R&D

We model the interaction between the health authority and the manufacturer sequentially. The timing of the model is as follows. Given the authorization policy, the health authority first sets the pricing policy (*p*_*l*_, *p*_*h*_). Then, the firm learns the values of *δ* and *I*. If $\delta \ge \hat{\delta}$ the firm decides whether to apply for authorization (it knows that the treatment will be authorized) or to undertake the R&D investment. If the firm carries out the investment, it finds the biomarker. The health authority administers the corresponding test to all patients, and the patients identified as responders are treated. If the firm does not undertake the investment, the treatment is authorized, and all patients are treated. If $\delta <\hat{\delta}$, the firm either undertakes R&D or the treatment is not marketed. As is usual, we use backward induction to characterize the optimal pricing policy. In other words, we characterize the subgame perfect equilibrium of the game. The timing of the model is depicted in Fig. 1.

## Results

In this section we characterize the optimal pricing policy. We analyze first the firm’s decision contingent on the pricing policy previously set by the health authority and then, we determine the price when the firm carries out the R&D investment and the price when personalized medicine is not implemented.

### The Firm’s decision

Given the pricing policy, once the firm knows *δ* and the cost of the R&D investment *I*, it must decide whether to undertake the R&D investment or to apply for market authorization whenever this option is feasible (if $\delta \ge \hat{\delta}\Big)$. If it does undertake it, the treatment is only administered to those identified by the test as responders. Its expected profits are then *sδ*(*p*_*h*_ − *c*) − c(1 − δ)(1 − e) − *I*. The firm expects to treat *sδ* + (1 − *δ*)(1 − *e*) patients, but only *sδ* are cured. The firm does not receive any payment for the treatments administered to (1 − *δ*)(1 − *e*) patients who are misidentified non-responders. When the firm does not undertake the R&D investment, the treatment is authorized and administered to all patients. The firm’s profits are *δp*_*l*_ − *c*. In this case, the firm is only paid when the patients respond to the treatment. Hence, when applying for market authorization is a feasible strategy, the firm undertakes R&D if this strategy yields expected profits higher than the profits from market authorization:

1 $$ {\displaystyle \begin{array}{c} s\delta \left({p}_h-c\right)-\mathrm{c}\left(1-\updelta \right)\left(1-\mathrm{e}\right)-I\ge \max\ \left(\updelta {p}_l-c,0\right)\\ {}\Downarrow \\ {} s\delta \left({p}_h-c\right)-\mathrm{c}\left(1-\updelta \right)\left(1-\mathrm{e}\right)-\max\ \left(\updelta {p}_l-c,0\right)\ge I\end{array}} $$

The firm will carry out the R&D investment if investment costs are sufficiently low. By letting *I*(*p*_*h*_, *p*_*l*_) denote the left-hand side of (1), the probability that the firm undertakes the R&D investment is *F*(*I*(*p*_*h*_, *p*_*l*_)).

When market authorization is not possible ($\delta <\hat{\delta}$), the firm will only sell the treatment if the patients are stratified. The firm undertakes R&D if:

2 $$ s\delta \left({p}_h-c\right)-\mathrm{c}\left(1-\updelta \right)\left(1-\mathrm{e}\right)-\mathrm{I}\ge 0\Longrightarrow s\delta \left({p}_h-c\right)-\mathrm{c}\left(1-\updelta \right)\left(1-\mathrm{e}\right)\ge I $$

By letting *I*(*p*_*h*_) denote the left-hand side of (2), the probability that the firm undertakes R&D when market authorization is not feasible is *F*(*I*(*p*_*h*_)).

### The health authority’s decision

In the first stage, the health authority chooses the pricing policy (*p*_*l*_, *p*_*h*_) to maximize expected net health benefits. When the patients are stratified, the expected net health benefits are *sδ*(*B* − *p*_*h*_) − (1 − *δ*)(1 − *e*)*L* − *t*. The new treatment will be only administered to the patients identified as responders by the test, and the health authority pays *p*_*h*_ for each cured patient. There will be (1 − *δ*)(1 − *e*)*L* patients identified as responders by the test who do not respond to the treatment (false positives), and experience adverse effects. Note that the test has to be administered to all patients. If the firm does not undertake the R&D investment, the net health benefits are *δ*(*B* − *p*_*l*_) − (1 − *δ*)*L*. The health authority pays *p*_*l*_ only for each responder, and non-responders experience adverse events. Thus, when both strategies are feasible, stratifying the patient population and applying for market authorization without R&D investment (e.g. if $\delta \ge \hat{\delta}\Big)$, the expected net health benefits are given by:

$$ F\left(I\left({p}_h,{p}_l\right)\right)\left( s\delta \left(B-{p}_h\right)-\left(1-\delta \right)\left(1-e\right)L-t\right)+\left[1-F\left(I\left({p}_h,{p}_l\right)\right)\right]\left(\delta \left(B-{p}_l\right)-\left(1-\delta \right)L\right) $$

A necessary condition for the firm to apply for authorization is *δp*_*l*_ − *c* ≥ 0. However, it may happen that, depending on *δ*, the health authority ends up with non-positive payoffs even when it chooses the lowest *p*_*l*_ compatible with non-negative profits for the firm:

$$ \delta B-c-\left(1-\delta \right)L<0\Longrightarrow \delta <\overset{\sim }{\delta } $$

where $\overset{\sim }{\delta }=\frac{L+c}{B+L}$. It follows that for $\delta <\overset{\sim }{\delta }$, unless the firm carries out the R&D investment, the health authority prefers the treatment not to be marketed. Given the assumptions in the model, we have $\overset{\sim }{\delta }>\hat{\delta}$. Thus, although the treatment would be authorized for $\delta \in \Big[\hat{\delta},\overset{\sim }{\delta }$), it is not worth the health authority giving the treatment to all patients. Therefore, it will choose any price *p*_*l*_ such that *δp*_*l*_ − *c* < 0 to discourage the firm from applying for market authorization. Hence, for $\delta <\overset{\sim }{\delta }$, the expected net health benefits are *F*(*I*(*p*_*h*_))(*sδ*(*B* − *p*_*h*_) − (1 − *δ*)(1 − *e*)*L* − *t*). Then, the health authority solves:

$$ {\max}_{p_h}\ F\left(I\left({p}_h\right)\right)\left( s\delta \left(B-{p}_h\right)-\left(1-\delta \right)\left(1-e\right)L-t\right)\kern0.5em (P1) $$

For $\delta \ge \overset{\sim }{\delta }$, the firm must decide between carrying over the R&D investment or applying for market authorization. The health authority chooses the prices (*p*_*h*_, *p*_*l*_) to maximize the expected net health benefits:

$$ \underset{p_h,{p}_l}{\max }\ F\left(I\left({p}_h,{p}_l\right)\right)\left( s\delta \left(B-{p}_h\right)-\left(1-\delta \right)\left(1-e\right)L-t\right)+\left[1-F\left(I\left({p}_h,{p}_l\right)\right)\right]\left(\delta \left(B-{p}_l\right)-\left(1-\delta \right)L\right)=F\left(I\left({p}_h,{p}_l\right)\right)\left[ s\delta \left(B-{p}_h\right)+e\left(1-\delta \right)L-t-\delta \left(B-{p}_l\right)-t\right]+\delta \left(B-{p}_l\right)-\left(1-\delta \right)L\ (P2a) $$

$$ s.t.\delta {p}_l-c\ge 0 $$

The constraint states that the firm’s profits are non-negative if the new treatment is administered to all patients.

**Lemma 1** *The constraint is binding:* ${p}_l=\frac{c}{\delta }$ *.*

*Proof* Let us suppose that ${p}_l>\frac{c}{\delta }$. If *p*_*h*_ ≤ *c*, there will be no R&D, and the health authority can increase expected net health benefits by setting ${p}_l=\frac{c}{\delta }$. Thus, the solution to (*P*2*a*) cannot be ${p}_l>\frac{c}{\delta }$ and *p*_*h*_ ≤ *c*.

Let us suppose that, in the solution, ${p}_l>\frac{c}{\delta }$ and *p*_*h*_ > *c*. In this case, from (1) there will be R&D whenever *I* ≤ *δ*(*sp*_*h*_ − *p*_*l*_) + *c*(1 − (1 − *δ*)(1 − *e*) − *sδ*). The health authority can guarantee itself the same R&D probability choosing ${p}_l=\frac{c}{\delta }$ and a price ${p}_h^{\prime }$ such that:

$$ {\displaystyle \begin{array}{c}\delta \left({sp}_h-{p}_l\right)+c\left(1-\left(1-\delta \right)\left(1-e\right)- s\delta \right)=\delta {sp}_h^{\hbox{'}}-c\left[\left(1-\delta \right)\left(1-e\right)- s\delta \right]\\ {}\Downarrow \\ {}\delta {sp}_h-\delta {p}_l+c=\delta {sp}_h^{\hbox{'}}\end{array}} $$

As *p*_*l*_ − *c* >0, it follows that ${p}_h>{p}_h^{\prime }$. Thus, the health authority can increase the expected net health benefits by setting lower prices: ${p}_l=\frac{c}{\delta }$ and $c<{p}_h^{\prime }<{p}_h$. Notice that

$$ {p}_h^{\prime }={p}_h-\frac{p_l}{s}+\frac{c}{s\delta} $$

If *p*_*h*_ ≥ *p*_*l*_, ${p}_h^{\prime }>c$. If *p*_*h*_ < *p*_*l*_, ${p}_h^{\prime }$ is also greater than *c*. As *δ*(*sp*_*h*_ − *p*_*l*_) + *c*[1 − (1 − *δ*)(1 − *e*) − *δs*] > 0, we need ${p}_h>\frac{p_l}{s}-\frac{c}{s\delta}\left[1-\left(1-\delta \right)\left(1-e\right)-\delta s\right]$ to have a positive R&D probability. Thus, we can write:

$$ {p}_h^{\prime }={p}_h-\frac{p_l}{s}+\frac{c}{s\delta}>\frac{p_l}{s}-\frac{c}{s\delta}\left[1-\left(1-\delta \right)\left(1-e\right)-\delta s\right]-\frac{p_l}{s}+\frac{c}{s\delta}=c\Big[1+\frac{\left(1-\delta \right)\left(1-e\right)}{s\delta} $$]

Therefore, regardless of the value of *p*_*h*_, ${p}_l=\frac{c}{\delta }$ (Q.E.D.)

The health authority appropriates the whole surplus, leaving the firm with zero profits, if there is no personalized medicine, and the treatment is given to all patients. By doing that, it increases the probability that the firm undertakes R&D. The net health benefits for the health authority when there is no patient stratification are *δB* − *c* − (1 − *δ*)*L*.

After substituting *p*_*l*_ into the objective function of (*P*2*a*), we have:

$$ {\max}_{p_h}\ F\left(I\left({p}_h,{p}_l\right)\right)\ \left[ s\delta \left(B-{p}_h\right)+e\left(1-\delta \right)L-t-\delta B+c\right]+\delta B-c-\left(1-\delta \right)L(P2b) $$

In order to get closed form solutions, from now on, we assume that *I* follows a uniform distribution $I\sim U\Big(0,\overline{I}$) where, to avoid trivial solutions, the upper limit $\overline{I}$ is sufficiently high.

From (2), the problem (*P*1) for $\delta <\overset{\sim }{\delta }$, can be now written as:

$$ \underset{p_h}{\max}\left[\frac{s\delta \left({p}_h-c\right)-c\left(1-\delta \right)\left(1-e\right)}{\overline{I}}\right]\left[ s\delta \left(B-{p}_h\right)-\left(1-\delta \right)\left(1-e\right)L-t\right] $$

The solution to this problem is

$$ {p}_h=c+\frac{s\delta \left(B-c\right)-t-\left(1-\delta \right)\left(1-e\right)\left(L-c\right)}{2 s\delta} $$

as long as $\delta >\frac{t+\left(1-e\right)\left(L-c\right)}{s\left(B-c\right)+\left(1-e\right)\left(L-C\right)}=\underset{\_}{\delta }$. Otherwise, the treatment is not marketed. Given the assumptions, $\underset{\_}{\delta }<\overset{\sim }{\delta }$:

$$ \underset{\_}{\delta }<\overset{\sim }{\delta}\iff t\left(B+L\right)<\left(B-c\right)\left[\left(c+L\right)\left(e+s-1\right)+2c\left(1-e\right)\right] $$

It can be easily shown that the price grows with *e* and *s*. Also, the higher *e* and *s*, the lower $\underset{\_}{\delta }$.

We may have personalized treatments for all $\delta \in \left(\underset{\_}{\delta },\overset{\sim }{\delta}\right)$ as long as the investment costs are sufficiently low:

$$ I\le \frac{s\delta \left(B-c\right)-t-\left(1-\delta \right)\left(1-e\right)\left(L+c\right)}{2} $$

**Proposition 1** *Let* (*c* + *L*)(*B* − *c*)(*e* + *s* − 1) > *t*(*B* + *L*)*. There may be personalized medicine for* $\delta \in \Big(\underset{\_}{\delta },\overset{\sim }{\delta }$*) if the investment costs are low enough. The price if the firm carries out the R&D investment is* ${p}_h=c+\frac{s\delta \left(B-c\right)-t-\left(1-\delta \right)\left(1-e\right)\left(L-c\right)}{2 s\delta}$*. For* $\delta \le \underset{\_}{\delta }$*, the treatment is not marketed.*

The results in Proposition 1 are quite intuitive. They hold when the payoff to the health authority if the treatment is administered to all patients is negative. Notice that it may happen that the treatment, although it could be authorized from a health perspective, is not marketed if the firm does not personalize the treatment. In the real world, many oncology treatments are highly valuable from a health perspective but, from the perspective of the health authorities, they are very expensive when given to all patients. According to the results in Proposition 1, they would be only marketed if the patients are stratified.

Notice that the price *p*_*h*_ when the firm carries out the R&D investment increases with *δ*:

$$ \frac{d{p}_h}{d\delta}=\frac{t+\left(1-e\right)\left(L-c\right)}{2s{\delta}^2}>0 $$

The larger the population of responders, the higher the price. It will be advantageous to the health authority to provide personalized treatments as, otherwise, its net health benefits are nil as no patient is treated. Therefore, the price when there is personalized medicine grows with the proportion of responders.

Let us now focus on the situation when the firm is not constrained to only carry out the R&D investment to sell the treatment. For $\delta \ge \overset{\sim }{\delta }$, the problem (*P*2*b*) can be written as:

$$ \underset{p_h}{\max}\left[\frac{s\delta \left({p}_h-c\right)-c\left(1-\delta \right)\left(1-e\right)}{\overline{I}}\right]\left[ s\delta \left(B-{p}_h\right)+e\left(1-\delta \right)L-t-\delta B+c\right]+\delta B-c-\left(1-\delta \right)L $$

The solution to this problem is:

$$ {p}_h=c+\frac{\left(1-\delta \right)\left( eL+\left(2-e\right)c\right)-\delta \left(B-c\right)\left(1-s\right)-t}{2 s\delta} $$

as long as $\delta <\overline{\delta}=\frac{eL+\left(2-e\right)c-t}{eL+\left(2-e\right)c+\left(B-c\right)\left(1-s\right)}$. Otherwise, any *p*_*h*_ ≤ *c* would be optimal. In this case, there would be no R&D, and the treatment would be given to all patients. Given the assumptions, $\overset{\sim }{\delta }<\overline{\delta}$:

$$ \overset{\sim }{\delta }<\overline{\delta}\iff t\left(B+L\right)<\left(B-c\right)\left[\left(c+L\right)\left(e+s-1\right)+2c\left(1-e\right)\right] $$

It is easy to see that the price grows with *e*. Also, the higher *e* and *s*, the greater $\overline{\delta}$

For $\delta \ge \overline{\delta}$, there is no personalized medicine. The proportion of responders is relatively high, and the health authority prefers the treatment to be given to all patients despite the adverse effects. For $\delta \in \left[\overset{\sim }{\delta },\overline{\delta}\right)$, the health authority provides incentives to stratify the patient population. Stratification is implemented if the investment cost is low. Otherwise, the treatment is given to all patients. The price is above *c* if the firm carries out the investment.

The firm carries out the R&D investment to find the biomarker if its profits, at least, cover the investment costs:

$$ \frac{e\left(1-\delta \right)\left(c+L\right)-\delta \left(B-c\right)\left(1-s\right)-t}{2}\ge I $$

Thus, there will be personalized medicine if the investment costs are sufficiently low. The probability that the R&D investment is carried out is given by:

$$ \frac{e\left(1-\delta \right)\left(c+L\right)-\delta \left(B-c\right)\left(1-s\right)-t}{2\overline{I}} $$

The greater the adverse effects, the more likely there will be personalized medicine. As the proportion of responders and the cost of the test increase, the likelihood of adopting personalized medicine falls.

The next proposition states the main results for $\delta \ge \overset{\sim }{\delta }$.

**Proposition 2** *Let* (*c* + *L*)(*B* − *c*)(*e* + *s* − 1) > *t*(*B* + *L*)*. For* $\delta \in \Big[\overset{\sim }{\delta }$*,* $\overline{\delta}\Big)$*, there will be personalized medicine if the investment costs are low enough. The price if the firm carries out the R&D investment is* ${p}_h=c+\frac{\left(1-\delta \right)\left( eL+\left(2-e\right)c\right)-\delta \left(B-c\right)\left(1-s\right)-t}{2 s\delta}$*. Otherwise, the price is* ${p}_l=\frac{c}{\delta }$*. For* $\delta \ge \overline{\delta}$ *there is no personalized medicine, and the price is* ${p}_l=\frac{c}{\delta }$*.*

The results in Proposition 2 are intuitive. They hold when the treatment would be authorized if the firm applied for it. In this case, if the proportion of patients who respond to the new treatment is too high ($\delta \ge \overline{\delta}$), all patients are given the new treatment, and there is no personalized medicine. For intermediate values of *δ*, the firm undertakes the R&D investment if the investment costs are sufficiently low, and medicine will be personalized (responders are the only patients treated).

The price *p*_*h*_ decreases with *δ*:

$$ \frac{d{p}_h}{d\delta}=\frac{t-\left( eL+\left(2-e\right)c\right)}{2s{\delta}^2}<0 $$

Intuitively, the larger *δ* is, the lower the proportion of non-responders. Thus, stratification is a less attractive option. On the other hand, the health authority must pay for more treatments, so it reduces the price. In the real world, drug prices are decided either through a negotiation process or by the firm; thus, we should expect that the more effective the drug the higher the price. However, in our model, the health authority chooses the pricing policy to maximize its expected net benefits. Hence, it does not have incentives to reward the firm when the population of responders is larger.

Let us know compare the prices when $\delta \in \Big[\overset{\sim }{\delta }$*,* $\overline{\delta}\Big)$, and both strategies (either pursuing patient stratification or applying for authorization) are feasible for the firm.

$$ {p}_h-{p}_l=\frac{\left(1-\delta \right)\left[e\left(L-c\right)-2c\left(1-s\right)\right]-\delta \left(1-s\right)\left(B-c\right)-t}{2 s\delta}\gtreqless 0\iff \frac{e\left(L-c\right)+2c\left(1-s\right)-t}{e\left(L-c\right)+2c\left(1-s\right)+\left(1-s\right)\left(B-c\right)}\gtreqless \delta $$

Let $\overleftrightarrow{\delta}$ denote the left hand side of the above expression. If $\overset{\sim }{\delta }>\overleftrightarrow{\delta}$, then the price when the firm undertakes the R&D investment is lower than the price when the firm does not undertake it for all $\delta \in \Big[\overset{\sim }{\delta }$*,* $\overline{\delta}\Big)$*.* This condition holds when (*L* − *c*)(*B* − *c*)(*e* + *s* − 1) < *t*(*B* + *L*). Thus, if the price of the test is relatively high, then *p*_*h*_ < *p*_*l*_ for all $\delta \in \Big[\overset{\sim }{\delta }$*,* $\overline{\delta}\Big)$*.* When the price of the test is relatively low (that is, *t*(*B* + *L*) ≤ (*B* − *c*)(*L* − *c*)(*e* + *s* − 1)), then $\overset{\sim }{\delta}\le \overleftrightarrow{\delta}$. In such a case, *p*_*h*_ ≥ *p*_*l*_ for $\delta \in \Big[\overset{\sim }{\delta }$*,* $\overleftrightarrow{\delta}\Big]$ and *p*_*h*_ < *p*_*l*_ for $\delta \in \left(\ \overleftrightarrow{\delta},\overline{\delta}\right)$.

We could expect *p*_*h*_ to be larger than *p*_*l*_ to reward the firm for stratifying patients. However, this is not necessarily true, even if the health authority prefers the firm to carry out the R&D investment. The relationship between *p*_*h*_ and *p*_*l*_ depends on the price of the test, the scale of adverse effects and production costs. In a setting without asymmetric information where the cost of the investment is common knowledge, it does not necessarily follow that the price when the firm carries out the R&D investment is higher than the price when there is no patient stratification. It will depend on the cost of the investment. In the setting considered here, there is asymmetric information and the health authority designs the pricing policy to maximize expected net health benefits. Notice that patient stratification happens with a probability lower than one, even if the firm were rewarded with a price higher than the price when it sells the treatment to all patients. The health authority leaves the firm with zero profits when there is no patient stratification. By doing that, it increases the probability that the firm will carry out the R&D investment, and firm’s profits are positive when the R&D investment is undertaken. Notice that the health authority must pay both for the drug and the test when patients are stratified. Depending on how costly the test is, it may end up paying a higher or lower price (i.e. *p*_*h*_ can be lower or higher than *p*_*l*_). In the limiting case when the price of the test tends to zero, *p*_*h*_ is always larger than *p*_*l*_. For intermediate values, the price when the firm carries out the R&D investment can be higher or lower than the price when there is no patient stratification.

Proposition 3 below summarizes the main findings in Propositions 1 and 2, and parameterizes the pricing policy and the decisions by the proportion of responders *δ*.

**Proposition 3** *Let* (*c* + *L*)(*B* − *c*)(*e* + *s* − 1) > *t*(*B* + *L*)*. For* $\delta <\underset{\_}{\delta }$*, the treatment is not marketed. For* $\delta \in \Big[\underset{\_}{\delta }$*,* $\overset{\sim }{\delta}\Big)$*, there may be personalized medicine if the investment costs are low enough. The price if the firm carries out the R&D investment is* ${p}_h=c+\frac{s\delta \left(B-c\right)-t-\left(1-\delta \right)\left(1-e\right)\left(L-c\right)}{2 s\delta}$*. If there is no patient stratification, the treatment is not marketed. For* $\delta \in \Big[\overset{\sim }{\delta }$*,* $\overline{\delta}\Big)$*, there may be personalized medicine if the investment costs are low enough. The price if the firm carries out the R&D investment is* ${p}_h=c+\frac{\left(1-\delta \right)\left( eL+\left(2-e\right)c\right)-\delta \left(B-c\right)\left(1-s\right)-t}{2 s\delta}$*. Otherwise, the price is* ${p}_l=\frac{c}{\delta }$ *and all the patients are treated. For* $\delta \ge \overline{\delta}$ *there is no personalized medicine, and the price is* ${p}_l=\frac{c}{\delta }$*.*

**Fig. 2:**
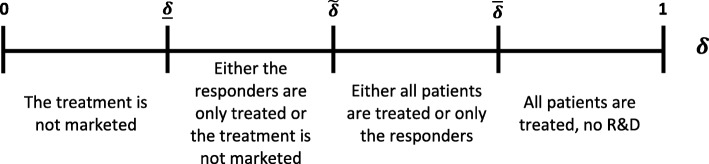
Decisions when (*c* + *L*)(*B* − *c*)(*e* + *s* − 1) > *t*(*B* + *L*). B = Economic value of health benefits, c = unit production cost, L = Economic value of adverse events, t = individual cost of administering the test, e = specificity of the test, s = sensitivity of the test

Figure 2 depicts, contingent on the values of *δ*, the different situations when (*c* + *L*)(*B* − *c*)(*e* + *s* − 1) > *t*(*B* + *L*).

So far, we have considered that the available test have specificities and sensitivities lower than one. The next corollaries present the results for the particular case of perfect tests.

**Corollary 1** *When s* = *e* = 1*, the threshold values* $\underset{\_}{\delta }$ *and* $\overline{\delta}$ *for the proportion of responders are:*

$$ \underset{\_}{\delta }=\frac{t}{\left(B-c\right)} $$

$$ \overline{\delta}=\frac{L+c-t}{L+c} $$

**Corollary 2** *When s* = *e* = 1*:*

$$ {p}_h-{p}_l=\frac{\left(1-\delta \right)\left(L-c\right)-t}{2\delta}\gtreqless 0\Longleftrightarrow 1-\frac{t}{L-c}\gtreqless \delta $$

**Corollary 3** *Let s* = *e* = 1*. Let* (*c* + *L*)(*B* − *c*) > *t*(*B* + *L*)*. For* $\delta <\underset{\_}{\delta }$*, the treatment is not marketed. For* $\delta \in \Big[\underset{\_}{\delta }$*,* $\overset{\sim }{\delta}\Big)$*, there may be personalized medicine if the investment costs are low enough. The price if the firm carries out the R&D investment is* ${p}_h=c+\frac{\delta \left(B-c\right)-t}{2\delta }$*. If there is no patient stratification, the treatment is not marketed. For* $\delta \in \Big[\overset{\sim }{\delta }$*,* $\overline{\delta}\Big)$*, there may be personalized medicine if the investment costs are low enough. The price if the firm carries out the R&D investment is* ${p}_h=c+\frac{\left(1-\delta \right)\left(L+\Big(c\right)-t}{2 s\delta}$*. Otherwise, the price is* ${p}_l=\frac{c}{\delta }$ *and all the patients are treated. For* $\delta \ge \overline{\delta}$ *there is no personalized medicine, and the price is* ${p}_l=\frac{c}{\delta }$*.*

**Fig. 3:**
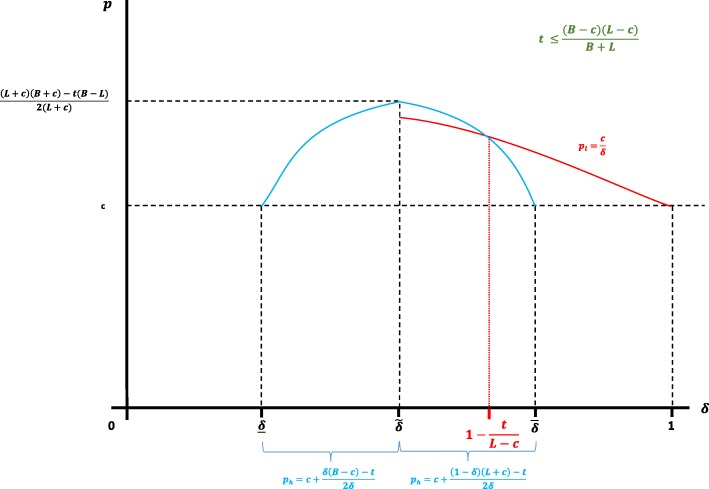
Prices (*p*_*l*_, *p*_*h*_) parameterized by the proportion of responders when *e* = *s* = 1

For the case *s* = *e* = 1, Fig. 3 depicts the prices set by health authority parameterised by the proportion of responders when (*c* + *L*)(*B* − *c*) > *t*(*B* + *L*).

## Discussion

The model provides a stylized description of the current interaction between pharmaceutical firms and health authorities when personalized medicine can be implemented. We have assumed (*c* + *L*)(*B* − *c*)(*e* + *s* − 1) > *t*(*B* + *L*) so that personalized medicine may be adopted when the health authority chooses the pricing policy. In the real world, health benefits are expected to outweigh side effects, and the cost of the test is usually low. Thus, we believe the case we have analyzed covers the most interesting situation. For the sake of clarity, we have excluded monitoring costs from the analysis; however, this variable does not qualitatively affect the analysis, although the threshold values of the proportion of responders for the adoption of personalized medicine would change accordingly.

Regarding the probability distribution *F*(*I*) of the investment cost, we have assumed that both the health authority and the firm know this distribution *ex-ante* although only the firm learns *ex-post* how costly its R&D investment has been. We start from the understanding that the firm always knows better than the health authority the cost of the R&D activities; in fact, it is well known that drug companies are reluctant to reveal cost information about these investments. For analytical purposes we have chosen a simple uniform distribution to derive closed forms solutions. Therefore, the results of the model are contingent on this assumption and should be accordingly revised if another distribution is selected. Also, for analytical tractability we have assumed that the efficacy of the new treatment is complete for the responders, although in real world this assumption may not hold completely. Nevertheless, as personalized medicine tailors the therapies to the best responders, we should expect the efficacy of the treatments to be quite high. For the sake of realism, we have considered that the available tests do not perfectly identify the responders (the specificity and the sensitivity are lower than one).

The results characterize the values for the proportion of responders for which personalized medicine may be implemented. Intuitively, when the proportion of responders is very high, from the health authority’s perspective, personalized medicine is less valuable. Likewise, when this proportion is very low, the cost of the test may offset the benefits and hence personalized medicine is not favourable either.

We have not considered the situation in which the firm has the power to set the price of the treatment. In this case, the firm will take the whole surplus regardless of the decision on R&D. Thus, the health authority will act as a price taker and will not have any instrument to promote the personalization of medicine. In the real world, this pricing policy may lead to price escalation and budgetary difficulties for healthcare systems.

A firm’s strategic decision about the best moment, either pre or post-approval, to carry out the R&D process is uncertain. In the model, we have considered only the pre-approval case. As we mention in the introduction, some authors [10] have analyzed the post-approval context, highlighting the role played by pay-for-performance schemes in the promotion of R&D activities to personalize treatments; in this model, the prices were given, and the analysis focused on how a reduction of the penalty the firms should pay when treatment fails could incentivize the search for additional information to stratify the patient population. In the current model, we assume full penalization for treatment failure, and characterize the pricing policy. Hence, it is not possible to compare the two models because they are formulated differently. Future research should address this topic and analyze which is the best moment for the R&D activities leading to patient stratification.

As observed in the results, the implementation of personalized medicine depends on the specific values of the parameters which define the threshold values for the proportion of responders. The larger *e* and *s*, the wider the range of values of the proportion of responders for which personalized medicine may be implemented. In other words, the better the quality of the available tests, the higher the possibility of having personalized treatments.

The model provides a useful framework for analyzing decision-making processes (prices and R&D to stratify the patient population) carried out by health authorities and pharmaceutical firms when dealing with treatment personalization. We have not considered a negotiation process between the firm and the health authority to set prices. Price and reimbursement schemes differ across jurisdictions. Pharmaceutical firms are initially free to set drug prices in some countries (e.g. the USA and Germany during the first year after launching), while in another ones (e.g. Spain, Italy and France), they are decided by health authorities after reviewing the proposals made by drug firms. It would be interesting to analyze the determination of prices throughout a bargaining process as the selection of the price-setting scheme could influence the choice when dealing with the adoption of personalized medicine. We hope to explore this issue in future research.

## Conclusions

Our findings show that pre-approval incentives (prices) to promote the personalized treatments depend on the specific characteristics of the disease, the efficacy of the treatment and the characteristics of the available tests. For relatively high values of the proportion of responders, health authorities have no interest in providing the firm with incentives to search for biomarkers. Personalized medicine may occur for intermediate values of the proportion of responders. Should the proportion of responders be relatively low, the firm will necessarily have to carry out the R&D investment to market the drug. For other values of the proportion of responders, the firm will have sales even if there is no patient stratification.

Contrary to what may be expected, the price when the firm invests in R&D to stratify the patient population is not necessarily higher than the price when such stratification does not take place. The relationship between both prices depends on the price of the test, the scale of adverse effects, the cost of producing the drug and the characteristics of the tests. Intuitively, the model shows that when the cost of the test is rather low, the price the health authority fixes when the firm carries out R&D investment is higher than the price when the treatment is administered to all patients. When tests are more expensive, then the opposite result holds. For intermediate values of the cost of the test, the relationship between both prices is contingent upon the efficacy of the treatment.

Personalization of medical treatments is a strategic decision for pharmaceutical firms. They have to invest in additional resources to carry out R&D activities to find the biomarkers to stratify the target population. Simultaneously, in order to maximize net health benefits, it is in the health authority’s interest to reduce the side effects of administering drugs to patients who will unlikely respond to treatments as well as to save drug costs. We have developed a theoretical analytical model to better understand the relationships between both agents when adopting these strategic decisions. This stylized model gives an intuitive idea about what to expect when the possibility of personalizing treatments becomes a strategic decision for the stakeholders.

## Data Availability

Not applicable

## Abbreviations

FOLFIRI: Folinic acid, fluorouracil, irinotecan
R&D: Research and development

## Footnotes

1 *B* and *L* must be interpreted as the economic values of health benefits and adverse events.

2 A biomarker indicates a change in expression or state of a protein that correlates with the susceptibility of the disease to a given treatment.

3 Strictly speaking, a non-treated patient derives some disutility from being ill. To save notation, we assume that the disutility is zero. The results qualitatively do not change.

4 The analysis of the case *t*(*B* + *L*) ≥ (*B* − *c*)(*L* + *c*)(*e* + *s* − 1) is available upon request. When the price of the test is relatively high, it can be shown that the treatment, if marketed, is given to all patients, and there is no personalized medicine.
